# plot2groups: an R package to plot scatter points for two groups of values

**DOI:** 10.1186/1751-0473-9-23

**Published:** 2014-12-03

**Authors:** Yong Xu, Fuquan Zhang, Guoqiang Wang, Hongbao Cao, Zaohuo Cheng, Yin Yao Shugart

**Affiliations:** Department of Psychiatry, First Hospital of Shanxi Medical University, Taiyuan, China; Wuxi Mental Health Center of Nanjing Medical University, Wuxi, Jiangsu Province China; Unit on Statistical Genomics, Intramural Research Program, National Institute of Mental Health, National Institutes of Health, Bethesda, Maryland USA

**Keywords:** R package, Plot2groups, Scatter point

## Abstract

**Background:**

Researchers usually employ bar graphs to show two groups of data, which can be easily manipulated to yield false impressions. To some extent, scatterplot can retain the real data values and the spread of the data. However, for groups of numeric data, scatterplot may cause over-plotting problems. As a result, many values all stack on top of each other.

**Results:**

We recently implemented an R package, plot2groups, to plot scatter points for two groups values, jittering the adjacent points side by side to avoid overlapping in the plot. The functions simultaneously calculate a P value of two group t- or rank-test and incorporated the P value into the plot.

**Conclusions:**

plot2groups is a simple and flexible software package which can be used to visualize two groups of values within the statistical programming environment R.

## Background

Comparing two groups of values is one of most common task faced by researchers. Visualizing these data in a graph may provide a clear and intuitive impression for the reader. Currently, bar graph is one of the most common methods of communicating statistical information—particularly, measures of central tendency, such as the mean, however, graphical asymmetry of bar graph gives rise to a corresponding cognitive asymmetry [1]. In addition, they fail to reveal key properties of the data, such as the exact number of observations, the outliers, and the distribution of the data. Thus bar graphs can be easily manipulated to yield false impressions.

Scatterplot is one of most commonly used strategy for visual representation of the relationship between two factors of the experiment. The advantages of scatterplot include retaining exact data values and sample size, showing minimum/maximum and outliers of the data. Scatterplot typically requires that data on both axes should be continuous. For groups of numeric values, one axis (typically the X axis) is discrete values representing categories. Plotting this kind of data can cause over-plotting problems so there are many similar values all stacked on top of each other. This makes it difficult to observe the full quantity of values in the dataset.

To address this issue, it is desirable to stagger the overlapping values side by side on the X axis. R packages ggplot2 can jitter the position of overlapped points when plotting categorical data [2]. However, it is not easy for common users to master its distinctive grammar. Thus, we built user-friendly functions to create such a plot by calling ggplot2.

### Implementation

The plot2groups package contains two functions, plot2 and plot2f. The main function plot2 is as follows:

plot2 (df, size, color, …).

The function takes a two-step procedure. First, the function carries out a two sample t- or rank-test to yield a P value; then, it plots a scatterplot for the data by calling the ggplot2 plotting system, incorporating the P value into the text of X label and an average bar into each group. In the plot2 function, parameter ‘df’ is a two-column data frame, the first column is numeric values, the second column is character or numeric vectors indicating two groups; parameter ‘size’ controls the size of the dots; and parameter ‘color’ is a two-string vector defining the color of the two groups.

## Results and discussion

We made two plots for the build-in dataset in the plot2groups package, which involved blood mRNA levels of the DRD3 gene [GenBank: U25441] in 37 schizophrenia patients and 37 healthy controls [3]. First, we load the drd3 data and plot a traditional point graph for the dataset using the ggplot2 system.

**Figure 1:**
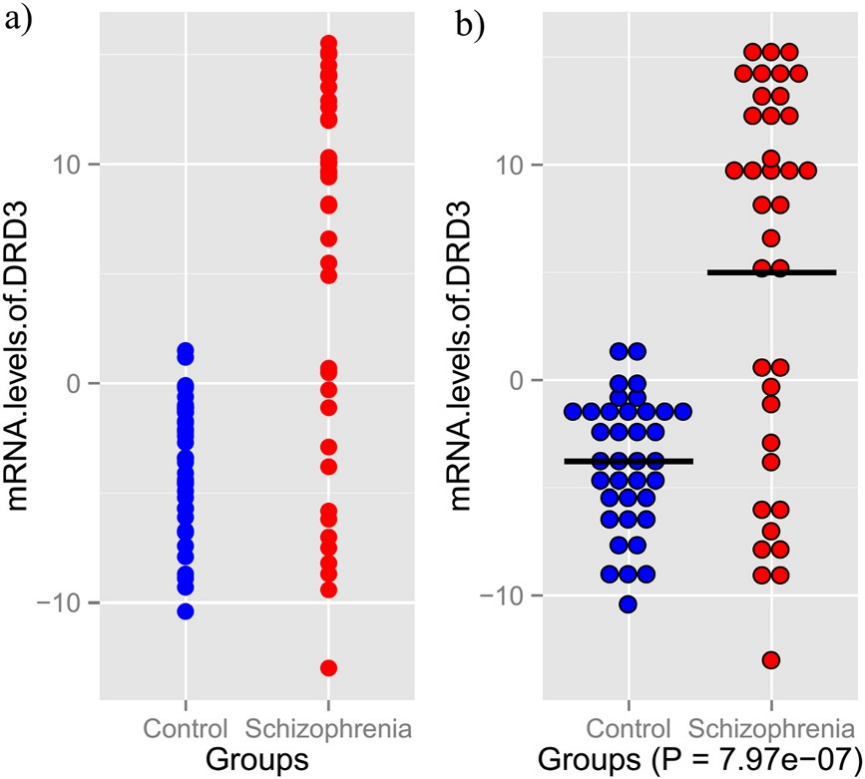
**Two example plots for the example data. a)** Traditional point plot for two groups of values. **b)** Scatterplot produced by plot2groups, and the bar is the average value for each group.

- > library(plot2groups)
- > data(drd3)
- > ggplot(drd3, aes(x = drd3, [2], y = drd3, [1])) + geom_point(color = c(rep(‘blue’, 37), rep(‘red’, 37)), size = 3) + xlab(names(drd3) [2]) + ylab(names(drd3) [1]).
- As can be seen in Figure 1 a), some points overlap with each other, especially in the ‘Control’ group, and it is impossible to discern the total number of sample.

To illustrate the functionality of plot2groups, we used the function ‘plot2’ to produce another graph on the drd3 data.

- > plot2(drd3)

As can be seen in Figure 1b, the package automatically lays adjacent points side by side, thus overcoming the overlapping amount the points. At the same, the package adds an average bar and the two-sample t-test P value into the graph.

plot2f is a similar function which takes a local data file as its first parameter.

Graphics are an important vehicle of communicating experimental data and results. However, many graphics fail to portray data at an appropriate level of details, presenting summary statistics rather than underlying distributions [4, 5]. Showing as much of the relevant underlying data as possible in the most meaningful, unbiased way, is a principle in data visualization. The plot2groups package provide easy-to-use functions to plot scatter points for two groups of values. It integrates statistical analysis and plotting function together to produce a graph for two group values. It overcomes the overlapping issue in a scatterplot for two groups of data, and incorporates some key properties of the data, including the P value and the average. One limitation is that the package applies to only two groups of values. In the future, we will extend it to multiple groups of data.

## Conclusions

plot2groups offers a friendly implementation for R users to plot scatter points for two groups of values. Future versions of the package will include more flexibility in terms of plotting parameters.

### Availability and requirements

The plot2groups package has been developed for the free statistical R environment (http://www.r-project.org) and runs under the major operating systems. The functions in the plot2groups package are accompanied by documentation files and simple examples to facilitate its use.

Project name: plot2groups

Project home page: http://cran.r-project.org/web/packages/plot2groups/index.html.

Operating system(s): Platform independent.

Programming language: R platform.

Other requirements: No.

License: GPL (≥3)

Any restrictions to use: It is available for free download.
